# Dichloridobis[3-(4-chloro­phen­yl)-2,*N*,*N*-trimethyl-2,3-di­hydro-1,2,4-oxa­diazole-5-amine-κ*N* ^4^]platinum(II)–4-chloro­benzaldehyde (1/1)

**DOI:** 10.1107/S1600536813017376

**Published:** 2013-07-10

**Authors:** Andreii S. Kritchenkov, Vladislav V. Gurzhiy, Nadezhda A. Bokach, Valentina A. Kalibabchuk

**Affiliations:** a Department of Chemistry, Saint Petersburg State University, Universitetsky Pr. 26, 198504 Stary Petergof, Russian Federation; b Department of Geology, Saint Petersburg State University, Universitetskaya Avenue 7/9, 199034, Saint Petersburg, Russian Federation; c O.O. Bohomolets National Medical University, Department of General Chemistry, Shevchenko blvd 13, 01004 Kiev, Ukraine

## Abstract

In the title 1:1 co-crystal, [PtCl_2_(C_11_H_14_ClN_3_O)_2_]·C_7_H_5_ClO, the coordination polyhedron of the Pt^II^ atom is slightly distorted square-planar with the chloride and 2,3-di­hydro-1,2,4-oxa­diazole ligands mutually *trans*, as the Pt atom lies on an inversion center. The 4-chloro­benzaldehyde mol­ecules are statistically disordered about an inversion centre with equal occupancies for the two positions. The Pt^II^ complex forms a three-dimensional structure through C—H⋯Cl and weaker C—H⋯O inter­actions with the 4-chloro­benzaldehyde mol­ecule.

## Related literature

For the synthesis of platinum complexes with 2,3-di­hydro-1,2,4-oxa­diazole ligands, see: Bokach *et al.* (2011 ▶); Kritchenkov *et al.* (2011 ▶). For related structures, see: Bokach *et al.* (2003 ▶, 2011 ▶); Kritchenkov *et al.* (2011 ▶); Bokach & Kukushkin (2006 ▶); Gushchin *et al.* (2008 ▶); Kuznetsov & Kukushkin (2006 ▶); Fritsky *et al.* (2006 ▶); Penkova *et al.* (2009 ▶). For standard bond lengths, see: see: Allen *et al.* (1987 ▶).
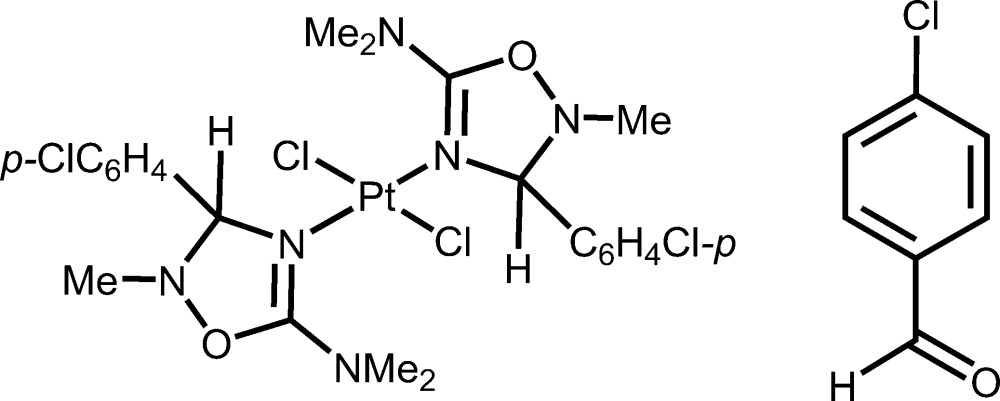


## Experimental

### 

#### Crystal data

[PtCl_2_(C_11_H_15_ClN_3_O)_2_]·C_7_H_5_ClO
*M* *_r_* = 887.97
Triclinic, 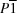

*a* = 8.46436 (18) Å
*b* = 9.38481 (19) Å
*c* = 11.4373 (3) Å
α = 101.0381 (18)°
β = 104.9553 (19)°
γ = 96.3847 (17)°
*V* = 849.07 (3) Å^3^
*Z* = 1
Mo *K*α radiation
μ = 4.57 mm^−1^
*T* = 100 K
0.17 × 0.11 × 0.09 mm

#### Data collection

Agilent Xcalibur Eos diffractometer
Absorption correction: multi-scan (*DENZO*/*SCALEPACK*; Otwinowski & Minor, 1997 ▶) *T*_min_ = 0.933, *T*_max_ = 1.000
13908 measured reflections
3892 independent reflections
3888 reflections with *I* > 2σ(*I*)
*R*_int_ = 0.036

#### Refinement

*R*[*F* ^2^ > 2σ(*F* ^2^)] = 0.022
*wR*(*F* ^2^) = 0.054
*S* = 1.06
3892 reflections
211 parameters
H-atom parameters constrained
Δρ_max_ = 1.63 e Å^−3^
Δρ_min_ = −1.21 e Å^−3^

### 

Data collection: *CrysAlis PRO* (Agilent, 2012 ▶); cell refinement: *CrysAlis PRO*; data reduction: *CrysAlis PRO*; program(s) used to solve structure: *SIR2004* (Burla *et al.*, 2005 ▶); program(s) used to refine structure: *SHELXL97* (Sheldrick, 2008 ▶); molecular graphics: *OLEX2* (Dolomanov *et al.*, 2009 ▶); software used to prepare material for publication: *SHELXL97*.

## Supplementary Material

Crystal structure: contains datablock(s) I, global. DOI: 10.1107/S1600536813017376/sj5329sup1.cif

Structure factors: contains datablock(s) I. DOI: 10.1107/S1600536813017376/sj5329Isup2.hkl

Additional supplementary materials:  crystallographic information; 3D view; checkCIF report

## Figures and Tables

**Table 1:** Hydrogen-bond geometry (Å, °)

*D*—H⋯*A*	*D*—H	H⋯*A*	*D*⋯*A*	*D*—H⋯*A*
C8—H8⋯O2^i^	0.96	2.58	3.378 (9)	141
C12—H12⋯Cl17^ii^	0.93	2.70	3.589 (4)	160

Symmetry codes: (i) 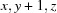; (ii) 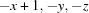.

**Fig. 1.**
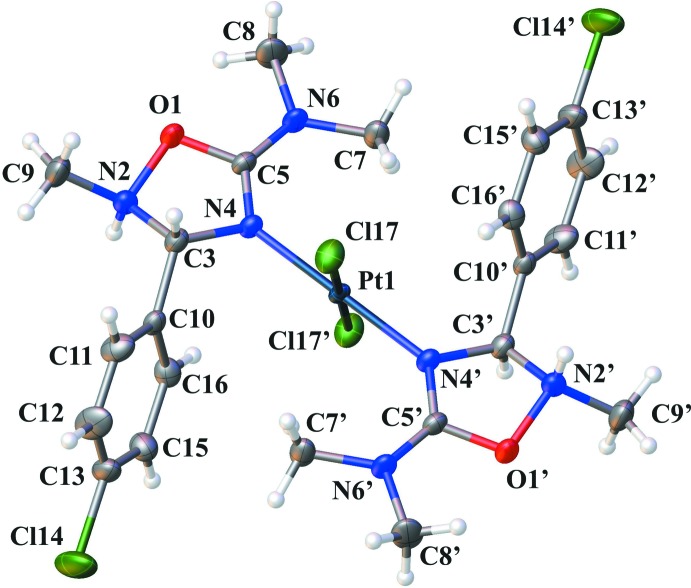
A view of the C22H30Cl4N6O2Pt complex in the structure of 1. Thermal ellipsoids are drawn at the 50% probability level. Pt atoms are blue, chlorine, carbon, nitrogen, and oxygen atoms are green, grey, pale blue, and red, respectively. Primed atoms are related to unprimed atoms by the symmetry operation -x+1, -y+1 -z.

**Fig. 2.**
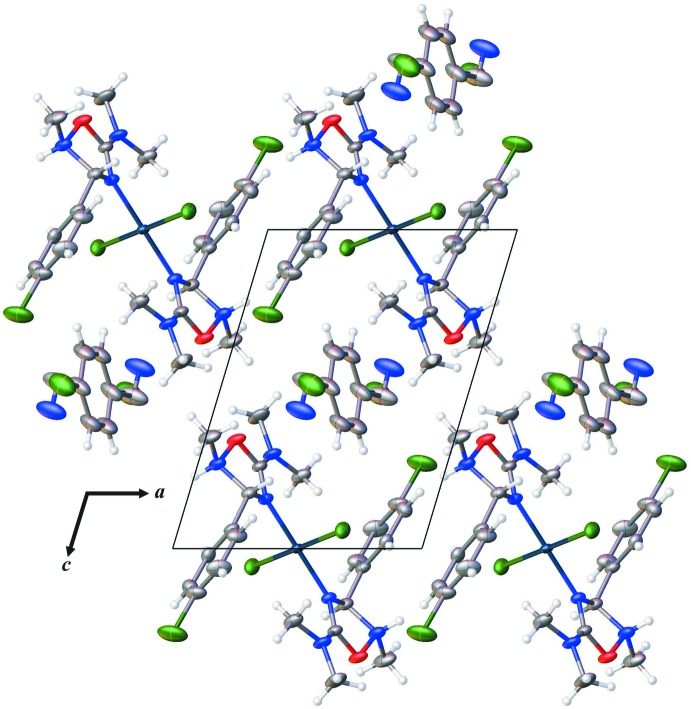
Crystal structure of the C22H30Cl4N6O2Pt.C7H5OCl associate, projection to the (010). Pt atoms are blue, chlorine, carbon,nitrogen, and oxygen atoms are green, grey, pale blue, and red, respectively.

## supplementary crystallographic information

### Comment

In the past decade, a great attention has been paid to metal-mediated cycloaddition (CA) of various dipoles to nitriles. Indeed, the activation of nitrile substrates by a metal center often results in promotion of CAs, which are not feasible in the so-called pure organic chemistry. In addition, metal-mediated CA represents an efficient route to free and/or coordinated heterocycles that could be either difficult to obtain or even inaccessible *via* metal-free protocols (Bokach *et al.*, 2011; Bokach & Kukushkin, 2006). Furthermore, an interest in platinum complexes with 2,3-dihydro-1,2,4-oxadiazole as a ligand is caused by their potential applications in medicine.

While 2,3-dihydro-1,2,4-oxadiazoles are known, examples of 5-dialkylamino-2,3-dihydro-1,2,4-oxadiazoles and, in particular, their metal complexes are still rare. Therefore, the synthesis of new complexes with 5-dialkylamino-2,3-dihydro-1,2,4-oxadiazole ligands and studies of their properties represent important tasks. As an amplification of our investigations of metal-mediated CA (Bokach *et al.*, 2011; Kuznetsov & Kukushkin, 2006) and the reactivity of metal-bound dialkylcyanamides (Kritchenkov *et al.*, 2011; Bokach *et al.*, 2003; Gushchin *et al.*, 2008), we have synthesized and characterized the title co-crystal and report its molecular and crystal structure here.

In 1, the complex molecule contains one crystallographically independent Pt atom that lies on an inversion center and is coordinated by two equivalent Cl^-^ anions and two N atoms (Fig. 1) of the heterocyclic ligands each of which are mutually *trans*. The Pt(1)–N(1) bond length is typical for (imine)Pt^II^ species (Allen *et al.*, 1987). The N(4)–C(5) (1.301 (4) Å) distance is characteristic for the N=C double bond (Fritsky *et al.*, 2006; Penkova *et al.*, 2009), while the N(4)–C(3) and N(2)–C(3) (1.476 (4) and 1.474 (4), respectively) are specific for the N–C single bonds (Allen *et al.*, 1987). Both asymmetric C(3) atoms in the heterocyclic ligands exhibit the same configuration (*RR*/*SS*). The p-chlorobenzaldehyde molecules are statistically disordered about an inversion centre with equal occupancies for the two half-occupied positions.

The platinum complexes are arranged in layers parallel to the (001) plane (Fig. 2). The *p-*chlorobenzaldehyde molecules occupy sites in between the layers of platinum(II) complexes.

### Experimental

The platinum complex was synthesized by a cycloaddition reaction between the complex trans-[PtCl_2_(NCNMe_2_)_2_] and the nitrone p-ClC6H4C(H)=N(O)Me as described previously (Kritchenkov *et al.*, 2011). Crystals of 1 were obtained from the reaction mixture by slow evaporation of the solvent (dichloromethane) at room temperature; p-chlorobenzaldehyde was generated in the reaction mixture by hydration of the nitrone in the undried solvent.

### Refinement

The carbon- and nitrogen-bound H atoms were placed in calculated positions and were included in the refinement in the riding model approximation, with U_iso_(H) set to 1.5U_eq_(C) and C–H 0.96 Å for the methyl groups, 1.2U_eq_(C) and C–H 0.98 Å for the tertiary CH groups, 1.2U_eq_(C) and C–H 0.93 Å for the carbon atoms of the benzene rings and aldehyde group, and 1.2U_eq_(N) and N–H 0.91 Å for the tertiary NH groups.

### Crystal data

**Table:**

[PtCl_2_(C_11_H_15_ClN_3_O)_2_]·C_7_H_5_ClO	*Z* = 1
*M**_r_* = 887.97	*F*(000) = 438
Triclinic, *P*1	*D*_x_ = 1.737 Mg m^−^^3^
*a* = 8.46436 (18) Å	Mo *K*α radiation, λ = 0.71073 Å
*b* = 9.38481 (19) Å	Cell parameters from 9801 reflections
*c* = 11.4373 (3) Å	θ = 2.5–31.7°
α = 101.0381 (18)°	µ = 4.57 mm^−^^1^
β = 104.9553 (19)°	*T* = 100 K
γ = 96.3847 (17)°	Prism, colourless
*V* = 849.07 (3) Å^3^	0.17 × 0.11 × 0.09 mm

### Data collection

**Table:**

Agilent Xcalibur Eos diffractometer	3892 independent reflections
Radiation source: Enhance (Mo) X-ray Source	3888 reflections with *I* > 2σ(*I*)
Graphite monochromator	*R*_int_ = 0.036
Detector resolution: 16.2096 pixels mm^-1^	θ_max_ = 27.5°, θ_min_ = 2.5°
ω scans	*h* = −10→10
Absorption correction: multi-scan (*DENZO*/*SCALEPACK*; Otwinowski & Minor, 1997)	*k* = −12→12
*T*_min_ = 0.933, *T*_max_ = 1.000	*l* = −14→14
13908 measured reflections	

### Refinement

**Table:**

Refinement on *F*^2^	Primary atom site location: structure-invariant direct methods
Least-squares matrix: full	Secondary atom site location: difference Fourier map
*R*[*F*^2^ > 2σ(*F*^2^)] = 0.022	Hydrogen site location: inferred from neighbouring sites
*wR*(*F*^2^) = 0.054	H-atom parameters constrained
*S* = 1.06	*w* = 1/[σ^2^(*F*_o_^2^) + (0.0326*P*)^2^] where *P* = (*F*_o_^2^ + 2*F*_c_^2^)/3
3892 reflections	(Δ/σ)_max_ < 0.001
211 parameters	Δρ_max_ = 1.63 e Å^−^^3^
0 restraints	Δρ_min_ = −1.21 e Å^−^^3^

### Special details

**Table:**

Geometry. All esds (except the esd in the dihedral angle between two l.s. planes) are estimated using the full covariance matrix. The cell esds are taken into account individually in the estimation of esds in distances, angles and torsion angles; correlations between esds in cell parameters are only used when they are defined by crystal symmetry. An approximate (isotropic) treatment of cell esds is used for estimating esds involving l.s. planes.
Refinement. Refinement of F^2^ against ALL reflections. The weighted R-factor wR and goodness of fit S are based on F^2^, conventional R-factors R are based on F, with F set to zero for negative F^2^. The threshold expression of F^2^ > 2sigma(F^2^) is used only for calculating R-factors(gt) etc. and is not relevant to the choice of reflections for refinement. R-factors based on F^2^ are statistically about twice as large as those based on F, and R- factors based on ALL data will be even larger.

### Fractional atomic coordinates and isotropic or equivalent isotropic displacement parameters (Å^2^)

**Table:**

	*x*	*y*	*z*	*U*_iso_*/*U*_eq_	Occ. (<1)
Pt1	0.5000	0.5000	0.0000	0.01562 (5)	
O1	0.8747 (3)	0.5755 (3)	0.34126 (19)	0.0321 (5)	
N2	0.9212 (3)	0.4486 (3)	0.2650 (2)	0.0290 (6)	
H2	0.9972	0.4786	0.2273	0.035*	
C3	0.7596 (4)	0.3810 (3)	0.1742 (3)	0.0243 (6)	
H3	0.6936	0.3203	0.2112	0.029*	
N4	0.6856 (3)	0.5130 (3)	0.1552 (2)	0.0209 (5)	
C5	0.7499 (4)	0.6134 (3)	0.2577 (3)	0.0240 (6)	
N6	0.7135 (3)	0.7450 (3)	0.2956 (2)	0.0281 (6)	
C7	0.5711 (4)	0.8001 (4)	0.2268 (3)	0.0334 (7)	
H7A	0.6050	0.8552	0.1723	0.050*	
H7B	0.5293	0.8627	0.2843	0.050*	
H7C	0.4856	0.7186	0.1788	0.050*	
C8	0.8131 (5)	0.8425 (4)	0.4142 (3)	0.0451 (10)	
H8A	0.7642	0.8272	0.4788	0.068*	
H8B	0.8163	0.9432	0.4077	0.068*	
H8C	0.9239	0.8209	0.4338	0.068*	
C9	0.9849 (5)	0.3574 (4)	0.3496 (3)	0.0452 (10)	
H9A	1.0813	0.4129	0.4134	0.068*	
H9B	1.0142	0.2721	0.3045	0.068*	
H9C	0.9012	0.3272	0.3869	0.068*	
C10	0.7869 (4)	0.2884 (3)	0.0606 (3)	0.0230 (6)	
C11	0.7575 (5)	0.1367 (4)	0.0400 (3)	0.0342 (7)	
H11	0.7156	0.0919	0.0941	0.041*	
C12	0.7907 (5)	0.0506 (4)	−0.0617 (3)	0.0434 (9)	
H12	0.7705	−0.0516	−0.0765	0.052*	
C13	0.8532 (5)	0.1191 (4)	−0.1391 (3)	0.0365 (8)	
Cl14	0.89823 (18)	0.01398 (12)	−0.26699 (9)	0.0619 (3)	
C15	0.8828 (4)	0.2708 (4)	−0.1219 (3)	0.0306 (7)	
H15	0.9243	0.3150	−0.1764	0.037*	
C16	0.8483 (4)	0.3547 (3)	−0.0206 (3)	0.0257 (6)	
H16	0.8667	0.4569	−0.0070	0.031*	
Cl17	0.34015 (9)	0.32493 (9)	0.05713 (7)	0.03003 (16)	
C19	0.5885 (5)	0.4816 (5)	0.6159 (4)	0.0510 (11)	
H19	0.6474	0.4686	0.6927	0.061*	
C20	0.5665 (6)	0.3740 (5)	0.5084 (4)	0.0493 (11)	
C18	0.4783 (5)	0.3921 (5)	0.3929 (4)	0.0479 (10)	
H18	0.4643	0.3190	0.3217	0.057*	
Cl21	0.6390 (5)	0.2036 (3)	0.5142 (4)	0.0640 (9)	0.50
C21	0.659 (3)	0.2527 (18)	0.522 (2)	0.081 (2)	0.50
H21	0.7255	0.2501	0.6005	0.097*	0.50
O2	0.6509 (12)	0.1563 (8)	0.4337 (7)	0.081 (2)	0.50

### Atomic displacement parameters (Å^2^)

**Table:**

	*U*^11^	*U*^22^	*U*^33^	*U*^12^	*U*^13^	*U*^23^
Pt1	0.01892 (8)	0.01346 (8)	0.01130 (7)	0.00185 (5)	0.00020 (5)	0.00142 (5)
O1	0.0370 (12)	0.0334 (13)	0.0173 (10)	0.0180 (10)	−0.0050 (9)	−0.0037 (9)
N2	0.0311 (13)	0.0305 (15)	0.0193 (12)	0.0135 (11)	−0.0004 (10)	−0.0033 (10)
C3	0.0280 (14)	0.0236 (15)	0.0188 (13)	0.0079 (12)	0.0013 (11)	0.0045 (11)
N4	0.0246 (12)	0.0176 (12)	0.0159 (11)	0.0040 (9)	−0.0003 (9)	0.0011 (9)
C5	0.0251 (14)	0.0276 (16)	0.0151 (13)	0.0071 (12)	0.0000 (11)	0.0017 (11)
N6	0.0340 (14)	0.0236 (13)	0.0170 (11)	0.0097 (11)	−0.0048 (10)	−0.0049 (10)
C7	0.0394 (18)	0.0260 (17)	0.0250 (15)	0.0147 (14)	−0.0038 (13)	−0.0047 (13)
C8	0.045 (2)	0.040 (2)	0.0297 (17)	0.0138 (16)	−0.0096 (15)	−0.0164 (15)
C9	0.053 (2)	0.048 (2)	0.0264 (17)	0.0291 (19)	−0.0062 (16)	0.0022 (16)
C10	0.0247 (14)	0.0207 (15)	0.0178 (13)	0.0064 (11)	−0.0022 (11)	0.0006 (11)
C11	0.051 (2)	0.0262 (17)	0.0257 (15)	0.0055 (15)	0.0084 (15)	0.0094 (13)
C12	0.074 (3)	0.0135 (16)	0.0358 (18)	0.0094 (16)	0.0061 (18)	0.0007 (14)
C13	0.054 (2)	0.0323 (19)	0.0212 (15)	0.0222 (16)	0.0059 (15)	−0.0002 (13)
Cl14	0.1140 (10)	0.0441 (6)	0.0333 (5)	0.0409 (6)	0.0248 (6)	0.0019 (4)
C15	0.0342 (16)	0.0305 (17)	0.0308 (16)	0.0112 (13)	0.0120 (14)	0.0087 (13)
C16	0.0235 (14)	0.0187 (15)	0.0309 (15)	0.0017 (11)	0.0043 (12)	0.0022 (12)
Cl17	0.0284 (4)	0.0283 (4)	0.0350 (4)	0.0005 (3)	0.0072 (3)	0.0162 (3)
C19	0.067 (3)	0.069 (3)	0.037 (2)	0.037 (2)	0.027 (2)	0.028 (2)
C20	0.071 (3)	0.058 (3)	0.044 (2)	0.042 (2)	0.037 (2)	0.028 (2)
C18	0.067 (3)	0.058 (3)	0.0366 (19)	0.031 (2)	0.0315 (19)	0.0178 (18)
Cl21	0.100 (2)	0.059 (2)	0.0583 (16)	0.0507 (19)	0.0399 (15)	0.0301 (19)
C21	0.145 (7)	0.062 (5)	0.059 (4)	0.064 (5)	0.043 (4)	0.023 (4)
O2	0.145 (7)	0.062 (5)	0.059 (4)	0.064 (5)	0.043 (4)	0.023 (4)

### Geometric parameters (Å, º)

**Table:**

Pt1—N4	2.018 (2)	C9—H9B	0.9600
Pt1—N4^i^	2.018 (2)	C9—H9C	0.9600
Pt1—Cl17	2.3087 (7)	C10—C11	1.381 (4)
Pt1—Cl17^i^	2.3087 (7)	C10—C16	1.385 (4)
O1—N2	1.490 (3)	C11—H11	0.9300
O1—C5	1.360 (3)	C11—C12	1.394 (5)
N2—H2	0.9100	C12—H12	0.9300
N2—C3	1.474 (4)	C12—C13	1.364 (5)
N2—C9	1.452 (4)	C13—Cl14	1.754 (3)
C3—H3	0.9800	C13—C15	1.385 (5)
C3—N4	1.476 (4)	C15—H15	0.9300
C3—C10	1.505 (4)	C15—C16	1.387 (4)
N4—C5	1.301 (4)	C16—H16	0.9300
C5—N6	1.325 (4)	C19—H19	0.9300
N6—C7	1.464 (4)	C19—C20	1.389 (6)
N6—C8	1.467 (4)	C19—C18^ii^	1.378 (6)
C7—H7A	0.9600	C20—C18	1.391 (5)
C7—H7B	0.9600	C20—Cl21	1.782 (5)
C7—H7C	0.9600	C20—C21	1.465 (18)
C8—H8A	0.9600	C18—C19^ii^	1.378 (6)
C8—H8B	0.9600	C18—H18	0.9300
C8—H8C	0.9600	C21—H21	0.9300
C9—H9A	0.9600	C21—O2	1.20 (2)
			
N4—Pt1—N4^i^	180.0	N2—C9—H9A	109.5
N4^i^—Pt1—Cl17	90.47 (7)	N2—C9—H9B	109.5
N4—Pt1—Cl17	89.53 (7)	N2—C9—H9C	109.5
N4^i^—Pt1—Cl17^i^	89.53 (7)	H9A—C9—H9B	109.5
N4—Pt1—Cl17^i^	90.47 (7)	H9A—C9—H9C	109.5
Cl17—Pt1—Cl17^i^	180.0	H9B—C9—H9C	109.5
C5—O1—N2	103.2 (2)	C11—C10—C3	119.9 (3)
O1—N2—H2	111.8	C11—C10—C16	119.8 (3)
C3—N2—O1	101.1 (2)	C16—C10—C3	120.2 (3)
C3—N2—H2	111.8	C10—C11—H11	119.9
C9—N2—O1	106.0 (2)	C10—C11—C12	120.1 (3)
C9—N2—H2	111.8	C12—C11—H11	119.9
C9—N2—C3	113.6 (3)	C11—C12—H12	120.6
N2—C3—H3	110.1	C13—C12—C11	118.8 (3)
N2—C3—N4	101.0 (2)	C13—C12—H12	120.6
N2—C3—C10	109.5 (2)	C12—C13—Cl14	119.9 (3)
N4—C3—H3	110.1	C12—C13—C15	122.6 (3)
N4—C3—C10	115.5 (2)	C15—C13—Cl14	117.5 (3)
C10—C3—H3	110.1	C13—C15—H15	121.1
C3—N4—Pt1	119.50 (17)	C13—C15—C16	117.8 (3)
C5—N4—Pt1	133.5 (2)	C16—C15—H15	121.1
C5—N4—C3	106.5 (2)	C10—C16—C15	120.9 (3)
N4—C5—O1	114.1 (3)	C10—C16—H16	119.6
N4—C5—N6	131.3 (3)	C15—C16—H16	119.6
N6—C5—O1	114.5 (2)	C20—C19—H19	120.4
C5—N6—C7	123.4 (2)	C18^ii^—C19—H19	120.4
C5—N6—C8	120.6 (3)	C18^ii^—C19—C20	119.1 (4)
C7—N6—C8	115.9 (3)	C19—C20—C18	121.0 (4)
N6—C7—H7A	109.5	C19—C20—Cl21	121.3 (3)
N6—C7—H7B	109.5	C19—C20—C21	116.0 (9)
N6—C7—H7C	109.5	C18—C20—Cl21	117.5 (4)
H7A—C7—H7B	109.5	C18—C20—C21	122.5 (9)
H7A—C7—H7C	109.5	C21—C20—Cl21	11.6 (10)
H7B—C7—H7C	109.5	C19^ii^—C18—C20	119.9 (4)
N6—C8—H8A	109.5	C19^ii^—C18—H18	120.1
N6—C8—H8B	109.5	C20—C18—H18	120.1
N6—C8—H8C	109.5	C20—C21—H21	119.7
H8A—C8—H8B	109.5	O2—C21—C20	120.5 (17)
H8A—C8—H8C	109.5	O2—C21—H21	119.7
H8B—C8—H8C	109.5		
			
Pt1—N4—C5—O1	179.0 (2)	C9—N2—C3—C10	−88.6 (3)
Pt1—N4—C5—N6	0.1 (5)	C10—C3—N4—Pt1	41.0 (3)
O1—N2—C3—N4	36.0 (3)	C10—C3—N4—C5	−146.2 (3)
O1—N2—C3—C10	158.3 (2)	C10—C11—C12—C13	0.5 (5)
O1—C5—N6—C7	−172.6 (3)	C11—C10—C16—C15	−0.7 (4)
O1—C5—N6—C8	4.9 (5)	C11—C12—C13—Cl14	179.4 (3)
N2—O1—C5—N4	16.2 (3)	C11—C12—C13—C15	−1.1 (6)
N2—O1—C5—N6	−164.7 (3)	C12—C13—C15—C16	0.7 (5)
N2—C3—N4—Pt1	159.05 (18)	C13—C15—C16—C10	0.2 (5)
N2—C3—N4—C5	−28.1 (3)	Cl14—C13—C15—C16	−179.7 (2)
N2—C3—C10—C11	105.8 (3)	C16—C10—C11—C12	0.4 (5)
N2—C3—C10—C16	−71.3 (3)	Cl17—Pt1—N4—C3	61.0 (2)
C3—N4—C5—O1	7.6 (3)	Cl17^i^—Pt1—N4—C3	−119.0 (2)
C3—N4—C5—N6	−171.3 (3)	Cl17^i^—Pt1—N4—C5	70.5 (3)
C3—C10—C11—C12	−176.7 (3)	Cl17—Pt1—N4—C5	−109.5 (3)
C3—C10—C16—C15	176.4 (3)	C19—C20—C18—C19^ii^	0.1 (8)
N4^i^—Pt1—N4—C3	15 (24)	C19—C20—C21—O2	−178.3 (15)
N4^i^—Pt1—N4—C5	−155 (24)	C18^ii^—C19—C20—C18	−0.1 (7)
N4—C3—C10—C11	−141.0 (3)	C18^ii^—C19—C20—Cl21	−176.3 (4)
N4—C3—C10—C16	41.9 (4)	C18^ii^—C19—C20—C21	172.0 (11)
N4—C5—N6—C7	6.3 (6)	C18—C20—C21—O2	−6 (3)
N4—C5—N6—C8	−176.2 (4)	Cl21—C20—C18—C19^ii^	176.4 (4)
C5—O1—N2—C3	−32.6 (3)	Cl21—C20—C21—O2	61 (4)
C5—O1—N2—C9	−151.3 (3)	C21—C20—C18—C19^ii^	−171.4 (11)
C9—N2—C3—N4	149.1 (3)		

Symmetry codes: (i) −*x*+1, −*y*+1, −*z*; (ii) −*x*+1, −*y*+1, −*z*+1.

### Hydrogen-bond geometry (Å, º)

**Table:**

*D*—H···*A*	*D*—H	H···*A*	*D*···*A*	*D*—H···*A*
C8—H8···O2^iii^	0.96	2.58	3.378 (9)	141
C12—H12···Cl17^iv^	0.93	2.70	3.589 (4)	160

Symmetry codes: (iii) *x*, *y*+1, *z*; (iv) −*x*+1, −*y*, −*z*.
